# microRNA-130b-3p Attenuates Septic Cardiomyopathy by Regulating the AMPK/mTOR Signaling Pathways and Directly Targeting ACSL4 against Ferroptosis

**DOI:** 10.7150/ijbs.82287

**Published:** 2023-08-15

**Authors:** Zhen Qi, Ruhui Liu, Haining Ju, Mengxi Huang, Zhe Li, Wei Li, Yongyi Wang

**Affiliations:** 1 Department of Cardiovascular Surgery, Renji Hospital, School of Medicine, Shanghai Jiao Tong University, Shanghai, China.; 2 Department of Cardiology, West China Second University Hospital, Sichuan University, Chengdu, Sichuan, China.; 3 Department of Cardiology, Seventh People's Hospital of Shanghai University of Traditional Chinese Medicine, Shanghai, China.; 4 Nanjing Drum Tower Hospital Clinical College of Traditional Chinese and Western Medicine, Nanjing University of Chinese Medicine, Nanjing, China.; 5 Department of Critical Care Medicine, Renji Hospital, School of Medicine, Shanghai Jiao Tong University, Shanghai, China.; 6 Cardiology Department of Ministry of Education, Sichuan University, Chengdu, Sichuan, China.

**Keywords:** microRNA-130b-3p, Ferroptosis, Autophagy, Septic cardiomyopathy, ACSL4, PRKAA1.

## Abstract

Ferroptosis is a newly identified type of programmed cell death that has been shown to contribute to the progression of septic cardiomyopathy. Although the role of miR-130b-3p as an oncogene that accelerates cancer progression by suppressing ferroptosis has been demonstrated, its role in the regulation of ferroptosis and cardiac injury in Lipopolysaccharide (LPS)-induced cardiomyopathy has not been fully clarified. In this study, we demonstrated that miR-130b-3p remarkably improved cardiac function and ameliorated morphological damage to heart tissue in LPS-induced mice. miR-130b-3p also improved cell viability and mitochondrial function and reduced the production of lipid ROS and ferroptosis in LPS-treated H9c2 cells. In addition, miR-130b-3p significantly upregulated GPX4 expression and suppressed ACSL4 activity in LPS-induced mouse heart tissue and H9c2 cells. Mechanistically, we used database analysis to locate miR-130b-3p and confirmed its inhibitory effects on the ferroptosis-related gene ACSL4 and autophagy-related gene PRKAA1 using a dual-luciferase reporter assay. In addition, we found that miR-130b-3p inhibited the activation of autophagy by downregulating the expression of the AMPK/mTOR signaling pathway. Meanwhile, our results show that RAPA (an autophagy activator) reverses the protective effect of miR-130b-3p mimic against LPS-induced ferroptosis, while CQ (an autophagy inhibitor) plays a facilitative role, suggesting that miR-130b-3p plays an important role in the development of ferroptosis by regulating autophagy *in vitro*. The findings reveal a novel function of miR-130b-3p in attenuating ferroptosis in cardiomyocytes, providing a new therapeutic target for ameliorating septic cardiomyopathy injury.

## Introduction

Sepsis is well-recognized as life-threatening multiorgan dysfunction caused by a dysregulated host immune response to infection 1. Despite significant advances in intensive care and life support techniques, hospitalization rates of sepsis have surpassed those for myocardial infarction and stroke in the West 2, 3. Existing epidemiologic studies have shown that the hospitalization mortality rate of patients with severe sepsis is as high as 25-30% 4, 5, and the hospitalization rate and mortality rate increase respectively by 8.2% and 5.6% per year 6. Septic cardiomyopathy is a common complication induced by sepsis, accounting for 70% of sepsis patients 7. Once cardiac dysfunction occurs, it may have a deleterious effect on tissue perfusion, the patient's physical condition deteriorates rapidly, and the mortality rate is as high as 50-60% 7. Septic cardiomyopathy is characterized by impaired left and/or right ventricular systolic and diastolic dysfunction, oxygen delivery, decreased LVEF (left ventricular ejection fraction), or primary myocardial cellular injury 7-9. Recent studies have demonstrated that the mechanisms of septic cardiomyopathy are involved in circulating mediators, molecular alterations, mitochondrial dysfunction, and cellular death 10, 11. Therefore, more aggressive approaches have been taken to improve cardiac function, which makes an independent contribution to the extent of poor outcomes. However, these strategies are often not ideal, and the underlying mechanisms of sepsis-induced myocardial injury remain unclear.

Ferroptosis is a novel iron-dependent form of regulated cell death characterized by mitochondrial shrinkage and increased mitochondrial membrane density, the accumulation of lipid hydroperoxides, and involvement of a unique set of genes, resulting in oxidative damage to cell membranes and is recognized to be distinct from apoptosis, necroptosis, and autophagy 12-14. Ferroptosis has been involved in various pathological processes associated with stroke 15, ischemia/reperfusion (I/R) injury 16, neoplastic diseases 17, and degenerative diseases 18. Recent studies have demonstrated that ferroptosis is a potential therapeutic target for various cardiovascular diseases (CVDs), including cardiomyopathy 19, myocardial infarction(MI) 20, myocardial I/R injury 21, 22, and Heart failure(HF) 23, 24. Acyl-CoA synthetase long-chain family member 4 (ACSL4), which is a key isozyme that regulates polyunsaturated fatty acids (PUFAs) metabolism, has been studied as a crucial factor in CVDs 25. ACSL4 has been recognized as a sensitive regulator of ferroptosis and a crucial contributor to the ferroptosis 26. Genetic and pharmacological inhibition of ACSL4 can trigger an anti-ferroptosis rescue pathway 27. The death of terminally differentiated cardiomyocytes is a vital pathogenic factor in the development of cardiac injury. It has been reported that various forms of cell death, including apoptosis, autophagy, necroptosis, and pyroptosis indicated in the septic cardiomyopathy 11, 28, 29. However, inhibiting either apoptosis or pyroptosis only partially improved the survival of LPS-induced cardiac cells 11, suggesting that another form of cell death may contribute to LPS-induced cardiotoxicity. A recent study has reported that Ferrostatin-1 (a ferroptosis inhibitor) alleviated LPS-induced cardiac injury, whereas Erastin and Sorafenib (ferroptosis inducers) aggravated, confirming that ferroptosis is activated in LPS-induced H9c2 cells 30.

Autophagy is a cellular catabolic process and the autophagic lysosomal degradation pathway is an important part of cardiac homeostasis 31. A recent study has reported that Cinnamaldehyde ameliorated LPS-induced cardiac dysfunction by inhibiting the production of reactive oxygen species and autophagy via the TLR-4 reduced nicotinamide adenine dinucleotide phosphate oxidase pathway 32. Rubicon knockout activates autophagy in cardiomyocytes through ER stress to improve myocardial injury and survival and increase cardiac output in septic mice 33.

On the other hand, a growing amount of evidence indicates that microRNAs (miRNAs, miRs) may play a crucial role in the septic cardiomyopathy pathophysiology 34, 35. miRNAs are evolutionarily conserved small non-coding RNAs of 22-24 nucleotides in length. They are induced in a variety of biological processes by binding to the 3'-untranslated region (3'-UTR) of the specific messenger RNAs (mRNA) to induce mRNA degradation and/or translational repression as post-transcriptional regulators of the gene expression 36. One member of the miR-130 family, miR-130b-3p has been found to play a vital role in multiple physiological and pathological processes, such as adipogenesis and Energy Metabolism, cancer, intervertebral disc degeneration, and cardiovascular disease 37-40. Recently, miR-130b-3p has been confirmed as one key molecule in sepsis, which can inhibit eCIRP-induced inflammation 41. However, the precise mechanism of how miR-130b-3p impacts the occurrence and development of septic cardiomyopathy is still not completely understood.

To our knowledge, the mechanism of miR-130b-3p in ferroptosis has never been reported in septic cardiomyopathy pathogenesis. Here, we demonstrated the critical role of miR-130b-3p in attenuating sepsis-induced cardiac dysfunction via the regulation of the AMPK/mTOR signaling pathways and directly targeting ACSL4 to suppress the ferroptosis in septic cardiomyopathy. The study has broad implications for miRNAs as prognostic biomarkers or tools to define therapeutic targeting molecular pathways.

## Materials and methods

### Reagents

Lipopolysaccharide (L2880) was purchased from Sigma (St. Louis, MO, United States). Erastin (≥ 99.42% purity), Ferrostatin-1 (Fer-1, ≥ 99.72% purity), Rapamycin (RAPA, ≥ 99.94% purity), and Chloroquine (CQ, ≥ 99.50% purity) were bought from MedChemExpress (Brea, CA, United States).

### Animals and animal models

All animal procedures performed were approved by the Animal Care and Use Committee of Renji Hospital, School of Medicine, Shanghai Jiao Tong University (Shanghai, China). Animal studies were reported in compliance with the Animal Research: Reporting *In Vivo* Experiments (ARRIVE) guidelines. All procedures were following the NIH Guide for the Care and Use of Laboratory Animals. Male C57BL/6J mice were purchased from the Institute of Laboratory Animal Science, Chinese Academy of Medical Sciences (Shanghai, China). The mice were housed at constant temperature (24°C ± 2°C) and humidity (50-60%) under a 12-hour light/dark cycle with free access to standard food and water. The sepsis-induced cardiomyopathy model was established by intraperitoneal injection of lipopolysaccharide (LPS) at a dose of 10 mg/kg (dissolved in sterile saline) for 12 hours as we previously study described 42.

### Cell culture and treatment

H9c2 cells were procured from Stem Cell Bank, Chinese Academy of Sciences, and cultured in high-glucose Dulbecco's modified Eagle medium supplemented with 10% fetal bovine serum and antibiotics (100 U/mL penicillin, 100 μg/ml streptomycin) at 37°C in an incubator containing 5% CO_2_ and 95% O_2_. H9c2 cells were grown on 6-well culture dishes until 80% confluence. After refreshing the medium, the cells were treated with LPS (10 μg/ml) for 12 h to create the LPS-induced cardiac injury cell model. Erastin (5 μM) was used to induce ferroptosis and Fer-1(2 μM) was used to suppress ferroptosis. Cells were collected for further analysis.

### Cell transfection

miR-130b-3p mimic, miR-130b-3p inhibitor, pCDH-ACSL4, and siRNA-AMPK were designed and synthesized by Bioegene (Shanghai, China). Lipofectamine 3000 (Invitrogen) was used to transfect the H9c2 cells. Briefly, the cells were cultured on 6-well plates until 60-70% confluence and then transfected with 30 pmol of negative control (NC)-mimic and miR-130b-3p-mimic; 30 pmol of NC-inhibitor and miR-130b-3p-inhibitor; 50 pmol of AMPK-siRNA; and 4 μg of pCDH-ACSL4 respectively. The detailed sequences are shown in **Supplemental Table S1.**

### Histological analysis

The murine heart tissues were fixed in 4% paraformaldehyde, embedded in paraffin, and transversely sectioned into 5 μm slices. For myofilament morphology and inflammatory cell infiltration assessment, the paraffin sections were stained with H & E solution (Beyotime Biotechnology) and analyzed under an optical microscope. Interstitial fibrosis was assessed by Masson's trichrome staining (Runnerbio biotech). The percentage of fibrosis is shown as the normalized ratio of the blue-stained area to the total area.

### Echocardiography

After 12 h of LPS injection, Cardiac function and structure were assessed using a high-resolution imaging system for small animals (Vevo3100 Imaging System, Visual Sonics, Japan). Briefly, mice were anesthetized with 1.5% isoflurane using ventilation equipment, fur has carefully removed from the left chest, and two- dimensional echocardiographic measurements were obtained. Left ventricular internal systolic dimension (LVIDs), left ventricular internal diastolic dimension (LVIDd), fractional shortening (FS), and ejection fraction (EF) were measured by Vevo3100 software (Visual Sonics) using M-mode tracing.

### Transmission electron microscopy

H9c2 cells and fresh myocardium (1 mm × 1 mm × 1 mm) were collected in sterile tubes and fixed with 2.5% glutaraldehyde solution overnight at 4 °C. Ultrathin sections were prepared after dehydration, permeation, and embedding. Hitachi HT-7800 TEM (Tokyo, Japan) was employed to observe the cardiomyocytes' mitochondrial size, membrane, and crest.

### Measurement of cell death and cell viability

Cell death was measured by PI staining coupled with microscopy. PI-staining positive cells were observed using fluorescence microscopy. Cell viability was measured using the Cell Counting Kit-8 (CCK 8) Assay Kit (Vazyme, China). Briefly, 5 × 10^3^ H9c2 cells/well were cultured in 96-well plates and incubated overnight. After the cells achieved 70% confluence, they were treated according to different experimental requirements. Subsequently, 10 μl CCK-8 solution was added to each well. After incubation at 37 °C for 2 h, the absorbance was measured at 450 nm.

### miRNA target analysis and Dual-luciferase reporter gene assay

To predict miR-130b-3p targets, PicTar, TargetScan, miRTarBase, and miRDB were used to scan the 3′-UTRs of mRNAs. Dual-luciferase reporter gene assay was performed to identify the putative binding sites (seed sequences) for miR-130b-3p in the 3′-UTR of ACSL4 and PRKAA1 mRNA. The wild-type (WT) ACSL4 and PRKAA1 3′-UTR as well as their mutant versions (MUT) were synthesized and inserted into a pmirGLO vector by Bioegene (Shanghai, China). DNA sequencing was used to verify the newly synthesized pmirGLO vectors. Briefly, H9c2 cells were seeded in 12-well plates and transfected with WT or MUT constructs, together with either miR-130b-3p mimics or negative control using Lipofectamine 3000 (Invitrogen). H9c2 Cells were harvested 48 h after transfection, and the luciferase activity was measured by Dual-Luciferase Reporter Assay Kit (Vazyme, China). The activity of firefly luciferase was normalized to Renilla luciferase.

### Reactive oxygen species and lipid peroxidation assays

The reactive oxygen species levels of H9c2 cells were measured using the ROS Assay Kit (Beyotime Biotechnology). In brief, H9c2 cells were plated in 12-well plates and incubated with LPS for 12 h. Then, DCFH-DA (10 μM) and Hoechst 33342 were added to the wells and incubated for 20 min at 37°C away from light. After washing twice with DMEM medium, H9c2 cells were observed using a fluorescence microscope. The relative intensity of DCFH-DA fluorescence was measured using Image J software (NIH, MD, United States). Lipid peroxidation was measured using C11-BODIPY 581/591 (Thermo Fisher Scientific). In brief, H9c2 cells were plated in 12-well plates and incubated with LPS for 12 h, then C11-BODIPY581/591 was added to the supernatant of the cell culture medium at a final concentration of 5 μmol/L. After incubation at 37 ◦C for 30 min in the dark and washing with PBS twice, H9c2 cells were observed using a fluorescence microscope.

### Measurement of the Mitochondrial Membrane Potential (ΔΨm)

The mitochondrial membrane potential (MMP) of H9c2 cells was measured using a JC-1 Mitochondria Membrane Potential Assay Kit purchased from Beyotime Biotechnology (Shanghai, China). All experimental procedures were performed according to the manufacturer's instructions. Briefly, H9c2 cells were washed twice with phosphate buffer saline (PBS), then incubated with JC-1 working solution at 37°C for 30 min away from light. After washing twice with JC-1 washing buffer and labeling cell nuclei with Hoechst 33342, images were observed by fluorescence microscopy. Red fluorescence indicates the normal mitochondrial potential, whereas green fluorescence indicates damaged mitochondrial potential. MMP was evaluated by the red-green ratio of fluorescence intensity. The relative fluorescence intensity was measured using Image J software (NIH, MD, United States).

### Western Blot analysis

Heart tissue and H9c2 cells were homogenized by radioimmunoprecipitation assay (RIPA) lysis buffer (Beyotime Biotechnology) supplemented with protease and phosphatase inhibitors. Protein concentration was quantified using the BCA Protein Assay Kit (Thermo Fisher Scientific, USA). An equivalent amount of protein extract was separated by SDS-PAGE and transferred to PVDF membranes (Millipore, USA). After blocking with 3% non-fat milk in Tris-buffered saline with Tween 20 at room temperature for 1 h, the membranes were incubated with primary antibodies at 4°C overnight. After that, they were probed with the corresponding HRP-conjugated secondary antibodies (Cell Signaling Technology) for 1 h at room temperature. Enhanced chemiluminescence (ECL, Vazyme, China) was used for immunoreactive detection), and densitometric quantification analysis was performed by Image J software (NIH, MD, United States). The following primary antibodies were utilized at indicated dilution: ACSL4 (1:1000, Santa Cruz Biotechnology, sc-365230), GPX4 (1:10000, Abcam, ab125066), GAPDH (1:1000, Cell Signaling Technology, mAb #5174S), p-AMPKα (1:1000, Cell Signaling Technology, mAb #2535S), AMPKα (1:1000, Cell Signaling Technology, mAb #5831S), p-mTOR (1:1000, Cell Signaling Technology, mAb #5536S), mTOR (1:1000, Cell Signaling Technology, mAb #2983S), SQSTM1(1:10000, Abcam, ab109012), Beclin1(1:1000, ABclonal, A17028), and LC3A/B (1:1000, Cell Signaling Technology, mAb #4108S).

### Quantitative real-time PCR (qRT-PCR)

Total RNA was isolated from heart tissue or H9c2 cells using EZ-press RNA Purification Kit (EZ-bioscience, United States) following the manufacturer's instructions. cDNA was then prepared in a 20 µL reaction volume using the HiScript III RT SuperMix for qPCR (gDNA wiper)/ miRNA 1st Strand cDNA Synthesis Kit (by stem-loop) (Vazyme, Nanjing, China) according to the manufacturer's instruction. Quantitative real-time PCR was performed using ChamQ SYBR GREEN Color qPCR Master Mix (Vazyme, Nanjing, China). GAPDH (for mRNA) and U6 (for miRNA) were used as the reference gene. The relative expression levels of mRNA and miRNA were quantified using the 2^-△△CT^ method. The sequencing information of primers in our research was given in **Supplementary Table S2**.

### Immunofluorescence staining

Heart sections were incubated with 10% BSA in PBS containing 0.1% Triton X-100 for 1 hour at room temperature, followed by overnight incubation at 4°C using the appropriate primary antibody. Anti-GFP (1:3000, Abcam, ab290) primary antibody was used. After incubation, the cells are washed three times with PBS containing 0.1% Triton X-100 at room temperature and then incubated with the appropriate fluorescence-labeled secondary antibody (Alexa Fluor® 488) (1:1000, Abcam, ab15077) for 1 hour at room temperature. DAPI was incubated to counterstain the cell nucleus, and then the mages were observed by fluorescence microscopy.

### MDA Assay

The lipid peroxidation MDA assay kit (Beyotime, Shanghai, China) was used to determine the level of lipid oxidative stress in cardiac tissue according to the manufacturer's instructions. Briefly, heart tissue was harvested, lysed, and centrifuged, and the supernatant was collected for MDA assay according to the manufacturer's instructions.

### Measurement of cardiac injury biomarkers

Blood was collected and centrifuged at 3,000 rpm for 15 min. The supernatant was collected. The levels of cardiac troponin (cTnI) and Creatine Kinase MB Isoenzyme (CK-MB) in serum were determined using the cTnI ELISA Kit and the CK-MB ELISA Kit (Yobibio, Shanghai, China) according to the manufacturer's instructions.

### Bioinformatics analysis

The published human RNA-sequencing (RNA-seq) raw data presented in this article is available in the Gene Expression Omnibus (GEO) repository (https://www.ncbi.nlm.nih.gov/geo/) and is accessible through GEO series accession number: GSE174507. Raw data were analyzed using the DESeq R package (1.36.0) based on the negative binomial distribution generalized linear model and the Benjamini-Hochberg method. The differentially expressed miRNAs were identified with cut-off |log2(Fold change) | ≥ 2, p-value < 0.05. Pathway enrichment analysis was performed using the clusterProfiler R package based on the Kyoto Encyclopedia of Genes and Genomes (KEGG) database (http://www.genome.jp/kegg/). p-value was calculated by a hypergeometric test. The top 12 enriched KEGG pathways with significance (P < 0.1) were visualized with bubble charts.

### Statistical analysis

All data were presented as mean ± standard deviation (SD). Statistical analyses were performed using GraphPad Prism 9.3 (GraphPad Software Inc., San Diego, CA, USA). Comparisons between the two groups were performed using Student's t-test and multiple groups were performed using one-way or two-way analysis of variance (ANOVA) with Tukey's posthoc test.* p* values < 0.05 were considered statistically significant.

## Results

### Overexpressing miR-130b-3p improved cardiac function in sepsis-induced cardiomyopathy mice

**FIGURE 1:**
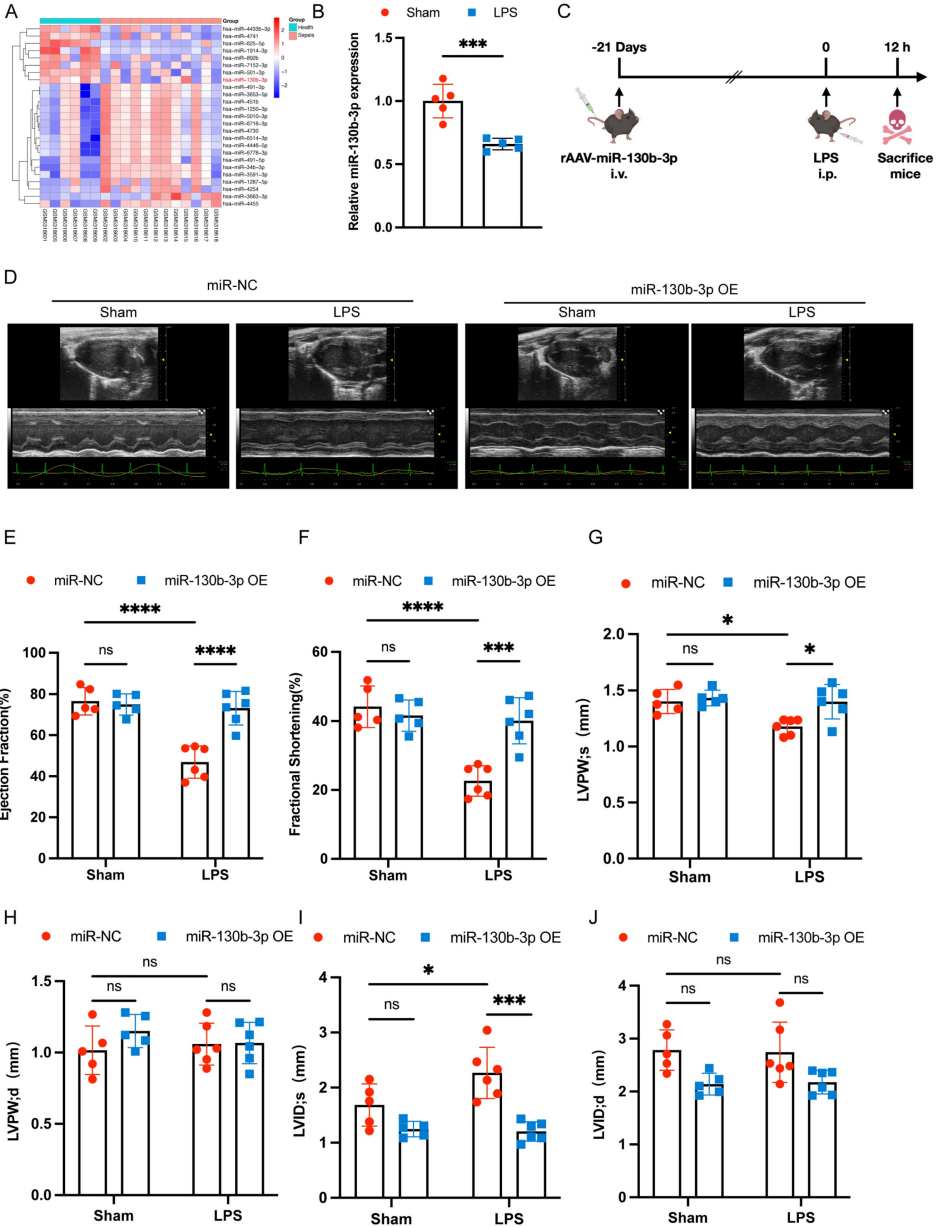
**| Overexpressing miR-130b-3p protected cardiac function in sepsis-induced cardiomyopathy mice. (A)** Heatmap of differentially expressed miRNAs in the Health group compared with the Sepsis group, |log_2_(Fold change)| ≥ 2, *p*-value < 0.05. **(B)** qRT-PCR analysis of miR-130b-3p in the myocardium of septic mice, n=5 per group. **(C)** Schematic diagram depicting the animal treatment procedure. **(D)** Representative images of echocardiography. Sham+miR-NC group n=5, Sham+miR-130b-3p OE n=5, LPS+ miR-NC group n=6, LPS+ miR-130b-3p OE n=6. **(E-J)** Echocardiographic analysis of **(E)** Ejection fraction (EF), **(F)** Fractional shortening (FS), **(G)** Left ventricular posterior wall systolic thickness (LVPWs), **(H)** Left ventricular posterior wall diastolic thickness (LVPWd), **(I)** Left ventricular internal systolic dimension (LVIDs), and **(J)** Left ventricular internal diastolic dimension (LVIDd). Data are expressed as mean ± SD. *p < 0.05, **p < 0.01, ***p < 0.001, ****p < 0.0001; ns: no significant difference.

To obtain novel biomarkers that serve as prompt indicators of septic cardiomyopathy and screen out the differentially expressed miRNAs (DEMs), we analyzed the GSE174507 of the GEO database. We found that the expression level of miR-130b-3p was significantly downregulated in the Peripheral blood of sepsis patients compared to healthy people (**Figure 1A**). At the same time, we found that the expression level of miR-130b-3p was also decreased in the hearts of mice with septic cardiomyopathy compared with the Sham group (**Figure 1B**). Therefore, we supposed that miR-130b-3p may play a vital role in the occurrence and development of septic cardiomyopathy. To overexpress the expression level of miR-130b-3p in the myocardium, the mice were injected with rAAV (recombinant adeno-associated virus)-miR-130b-3p through the tail vein for 21 days, and then LPS (10 mg/kg) was given intraperitoneally for 12h. (**Figure 1C**). RT-qPCR and immunofluorescence results showed that the expression level of miR-130b-3p was significantly upregulated in the myocardium of mice (**Figure S1A, B**). To investigate the effects of miR-130b-3p on cardiac function in the sepsis mice model, cardiac function parameters were evaluated by echocardiography (**Figure 1D**). The echocardiography results revealed that septic cardiomyopathy mice had significantly impaired cardiac function, as evidenced by the markedly reduced EF% (**Figure 1E**), FS% (**Figure 1F**), and LVPWs (**Figure 1G**) and markedly increased LVIDs (**Figure 1I**). miR-130b-3p OE markedly reversed these adverse effects. The groups found no significant differences between LVPWd (**Figure 1H**) and LVIDd (**Figure 1J**). Inversely, the expression level of miR-130b-3p was inhibited by using rAAV-miR-130b-3p Sponge (**Figure S1C**). The echocardiography results revealed that knocking down miR-130b-3p deteriorated cardiac function in sepsis-induced cardiomyopathy mice as evidenced by the markedly reduced EF% (**Figure S2B**) and FS% (**Figure S2C**). These results demonstrate that miR-130b-3p plays an important role and miR-130b-3p OE protected cardiac function in septic cardiomyopathy mice.

### Overexpressing miR-130b-3p prevented myocardial injury and ferroptosis in sepsis-induced cardiomyopathy mice

**FIGURE 2:**
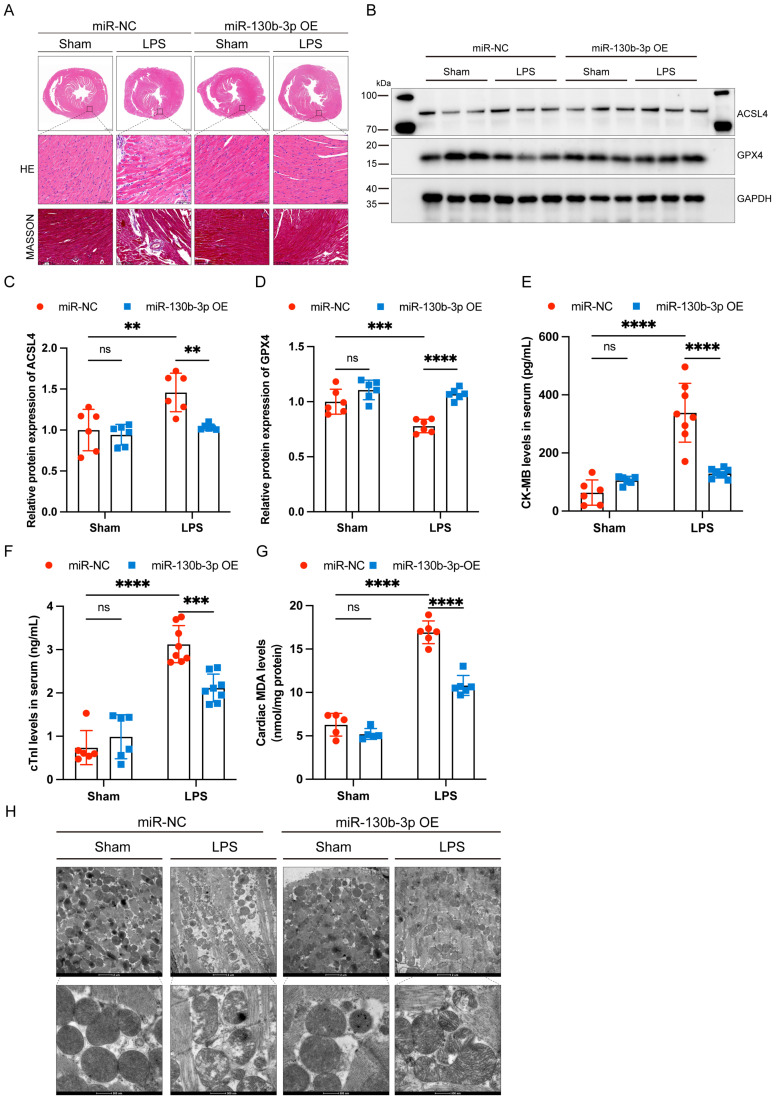
**| Overexpressing miR-130b-3p prevented myocardial injury and ferroptosis in sepsis-induced cardiomyopathy mice. (A)** Representative H&E staining and Masson trichrome staining images of myocardium from septic mice, n = 6. Scale bar, 100 μm. **(B)** Representative immunoblots and **(C-D)** densitometric quantification analysis of the protein expression levels of ACSL4 and GPX4 in cardiac tissue of septic mice, n=6. **(E)** Serum cardiac tissue creatine kinase isoenzyme (CK-MB) levels, n=6. **(F)** Serum cardiac troponin (cTnI) levels detected by ELISA, n = 6. **(G)** MDA levels of myocardium from septic mice, n = 6. **(H)** Representative images of TEM of myocardium from septic mice, n = 6. Scale bar, 500 nm. Data are expressed as mean ± SD. **p < 0.01, ***p < 0.001, ****p < 0.0001; ns: no significant difference.

Extensive inflammatory damage to the myocardium is a major pathological feature of septic cardiomyopathy. HE staining showed that in the LPS group cardiomyocytes were disorganized, edematous, and infiltrated with inflammatory cells, and miR-130b-3p overexpression reversed this adverse damage. Masson trichrome staining showed that a small amount of collagen deposition occurs around the blood vessels of the myocardium in LPS-treated mice, and overexpression of miR-130b-3p inhibited collagen deposition (**Figure 2A**). Western blot showed that ferroptosis-associated proteins GPX4 and ACSL4 were aberrantly expressed in the hearts of LPS-treated mice, and overexpression of miR-130b-3p downregulated the expression level of ACSL4 and upregulated the expression level of GPX4 (**Figure 2B-D**). Moreover, overexpression of miR-130b-3p inhibited the release of LPS-induced myocardial CK-MB and cTnI and protected against myocardial injury (**Figure 2E, F**). In the LPS group, MDA levels were highly expressed, while overexpression of miR-130b-3p suppressed MDA levels in the myocardium of septic mice (**Figure 2G**). Transmission electron microscopy showed that cardiac ultrastructural injury was more severe in the septic mice than in the control group, characterized by mitochondrial swelling, mitochondrial fission, vacuolar degeneration, and mitochondrial cristae rarefication. Importantly, Overexpression of miR-130b-3p alleviated the above-mentioned ultrastructural injuries in the LPS-induced myocardial mitochondrial damage (**Figure 2H**). Inversely, knocking down miR-130b-3p deteriorated myocardial injury and ferroptosis in sepsis-induced cardiomyopathy mice (**Figure S3**). The above results suggest that overexpression of miR-130b-3p protects against myocardial injury in septic cardiomyopathy as well as regulates ferroptosis.

### Inhibition of ferroptosis alleviated LPS-induced H9c2 cells injury

**FIGURE 3:**
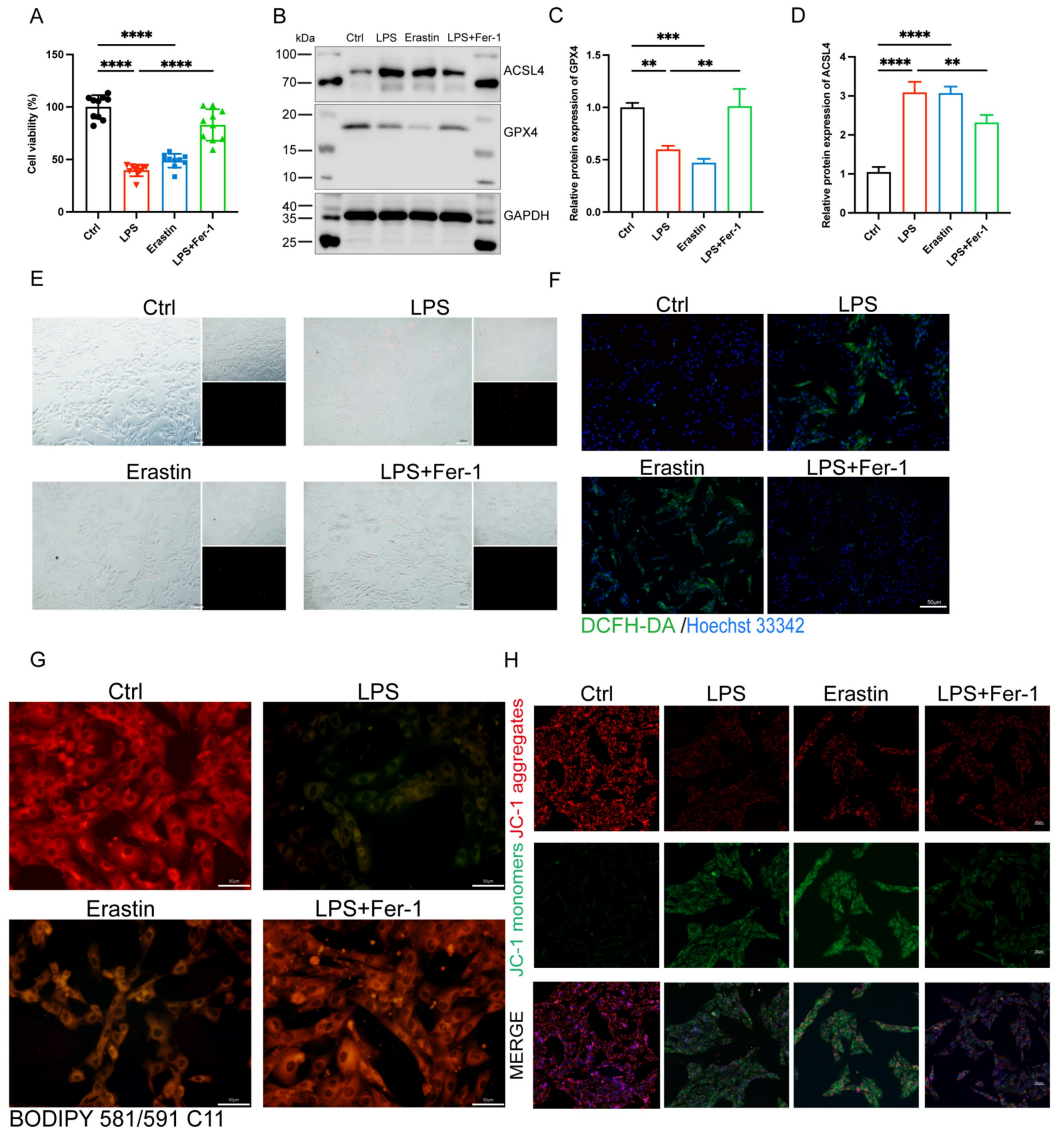
**| Inhibition of ferroptosis alleviated LPS-induced cardiomyocyte injury. (A)** Cell viability was detected by Cell Counting Kit-8 Assay (CCK8) in the indicated groups, n = 10. **(B)** Immunoblots analysis and **(C-D)** densitometric quantification of the protein expression levels of ACSL4 and GPX4 in LPS-induced H9c2 cells, n=3. **(E)** Representative images of microscopy (black and white: phase contract; red: PI staining), n=3. Scale bar, 100 μm. **(F)** Representative images of DCFH-DA/Hoechst 33342 staining, n=3. Scale bar, 50 μm. **(G)** Representative immunofluorescence staining images of lipid ROS in LPS-induced H9c2 cells via BODIPY 581/591 C11 probe, n=3. Scale bar, 50 μm. **(H)** Representative images of Mitochondrial membrane potential evaluated by JC-1 staining, n=3. Scale bar, 50 μm. Data are expressed as mean ± SD. **p < 0.01, ***p < 0.001, ****p < 0.0001; ns: no significant difference.

To further illuminated the adverse role of ferroptosis in septic cardiomyopathy, the H9c2 cells were stimulated with both LPS and Erastin (an inducer of ferroptosis) to establish a cell model, and Fer-1 was administered 2 h before the LPS challenge. We found that treatment with both LPS and Erastin reduced cell viability significantly, and Fer-1 restored cell viability in the LPS+Fer-1 group (**Figure 3A**). Western blot showed that the protein expression level of ASCL4 was significantly upregulated and GPX4 was significantly downregulated in the LPS and Erastin groups, and Fer-1 treatment significantly upregulated the expression of GPX4 and downregulated the expression of ACSL4 (**Figure 3B-D**). In addition, PI staining showed that both LPS and Erastin resulted in cell rupture, swelling, and necrosis, while Fer-1 treatment significantly reduced cell death (**Figure 3E**). We further found that, similar to Erastin, LPS treatment led to a significant increase in the levels of cellular ROS, while Fer-1 significantly reduced them and could inhibit the significantly increased lipid ROS levels after LPS treatment (**Figure 3F, G**). Meanwhile, LPS treatment significantly decreased the levels of mitochondrial membrane potential, while Fer-1 treatment reversed this adverse effect (**Figure 3H**). The above results indicate that LPS caused the occurrence of ferroptosis in H9c2 cells, while inhibition of ferroptosis could attenuate LPS-induced cell death.

### MiR-130b-3p alleviated ferroptosis in LPS-induced H9c2 cells

**FIGURE 4:**
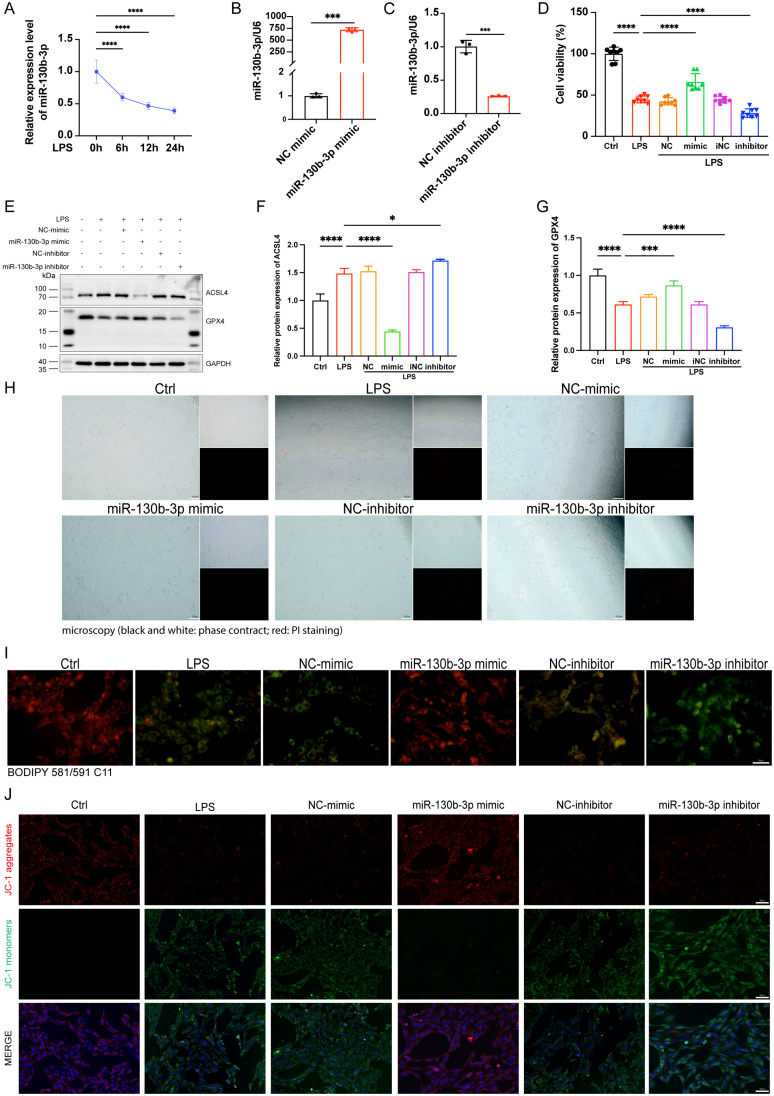
**| miR-130b-3p alleviated ferroptosis in LPS-induced cardiomyocytes. (A)** The H9c2 cells were treated with 10 ug/ml LPS for indicated time points. The expression levels of miR-130b-3p were determined by qRT-PCR, n=3. **(B-C)** The H9c2 cells were transfected with mimic-NC, miR-130b-3p mimic or inhibitor-NC, and miR-130b-3p inhibitor for 24 h. The expression levels of miR-130b-3p were determined by RT-qPCR, n=3. **(D)** Cell viability was detected by Cell Counting Kit-8 Assay (CCK8) in the indicated groups, n=8. **(E)** Immunoblots analysis and (F-G) densitometric quantification of the protein expression levels of ACSL4 and GPX4 in the indicated groups, n=3. **(H)** Representative images of microscopy (black and white: phase contract; red: PI staining), n=3. Scale bar, 100 μm. **(I)** Representative immunofluorescence staining images of lipid ROS in LPS-induced H9c2 cells via BODIPY 581/591 C11 probe, n=3. Scale bar, 50 μm. **(J)** Representative images of Mitochondrial membrane potential evaluated by JC-1 staining, n=3. Scale bar, 50 μm. Data are expressed as mean ± SD. *p < 0.05, ***p < 0.001, ****p < 0.0001; ns: no significant difference.

To investigate the function of miR-130b-3p in LPS-induced H9c2 cells, we treated H9c2 cells with LPS for 0, 6, 12, and 24 hours respectively. We found that the expression level of miR-130b-3p decreased gradually with the extension of LPS stimulation time (**Figure 4A**). We transfected miR-130b-3p mimic and inhibitor *in vitro* for the subsequent research. qRT-PCR results showed that miR-130b-3p mimic significantly elevated the expression level of miR-130b-3p, while miR-130b-3p inhibitor suppressed it (**Figure 4B, C**). CCK-8 assay revealed that LPS treatment of H9c2 cells for 12 h significantly decreased cell viability, and transfection of miR-130b-3p mimic significantly increased cell viability of LPS-stimulated H9c2 cells; transfection of miR-130b-3p inhibitor exacerbated LPS-induced H9c2 cell death (**Figure 4D**). As shown in (**Figure 4E-G**), LPS treatment caused abnormal expression levels of ferroptosis-related proteins ACSL4 and GPX4. miR-130b-3p mimic downregulated protein expression levels of ACSL4 and upregulated protein expression levels of GPX4. Meanwhile, we observed by PI staining as well as the white phase that LPS treatment caused H9c2 cell death, while miR-130b-3p mimic attenuated LPS-induced cell death, on the contrary, miR-130b-3p inhibitor aggravated LPS-induced H9c2 cell death (**Figure 4H**). Ferroptosis is characterized by excessive lipid peroxidation and mitochondria dysfunction. Abnormal alteration of mitochondrial membrane potential indicates mitochondrial damage and early warning of ferroptosis 19. As shown in (**Figure 4I**), LPS stimulation significantly increased the intracellular lipid peroxidation level in H9c2 cells, while miR-130b-3p mimic attenuated the accumulation of lipid ROS and conversely miR-130b-3p inhibitor exacerbated this detrimental damage. JC-1 staining showed that LPS treatment significantly decreased the level of mitochondrial membrane potential in H9c2 cells, while miR-130b-3p mimic significantly increased the mitochondrial membrane potential (**Figure 4J**). The above findings suggest that miR-130b-3p is involved in the process of ferroptosis in LPS-induced H9c2 cells cardiomyocytes, and miR-130b-3p mimic significantly attenuated the progression of ferroptosis in septic cardiomyocytes.

### ACSL4 and PRKAA1 are the direct targets of miR-130b-3p

**FIGURE 5:**
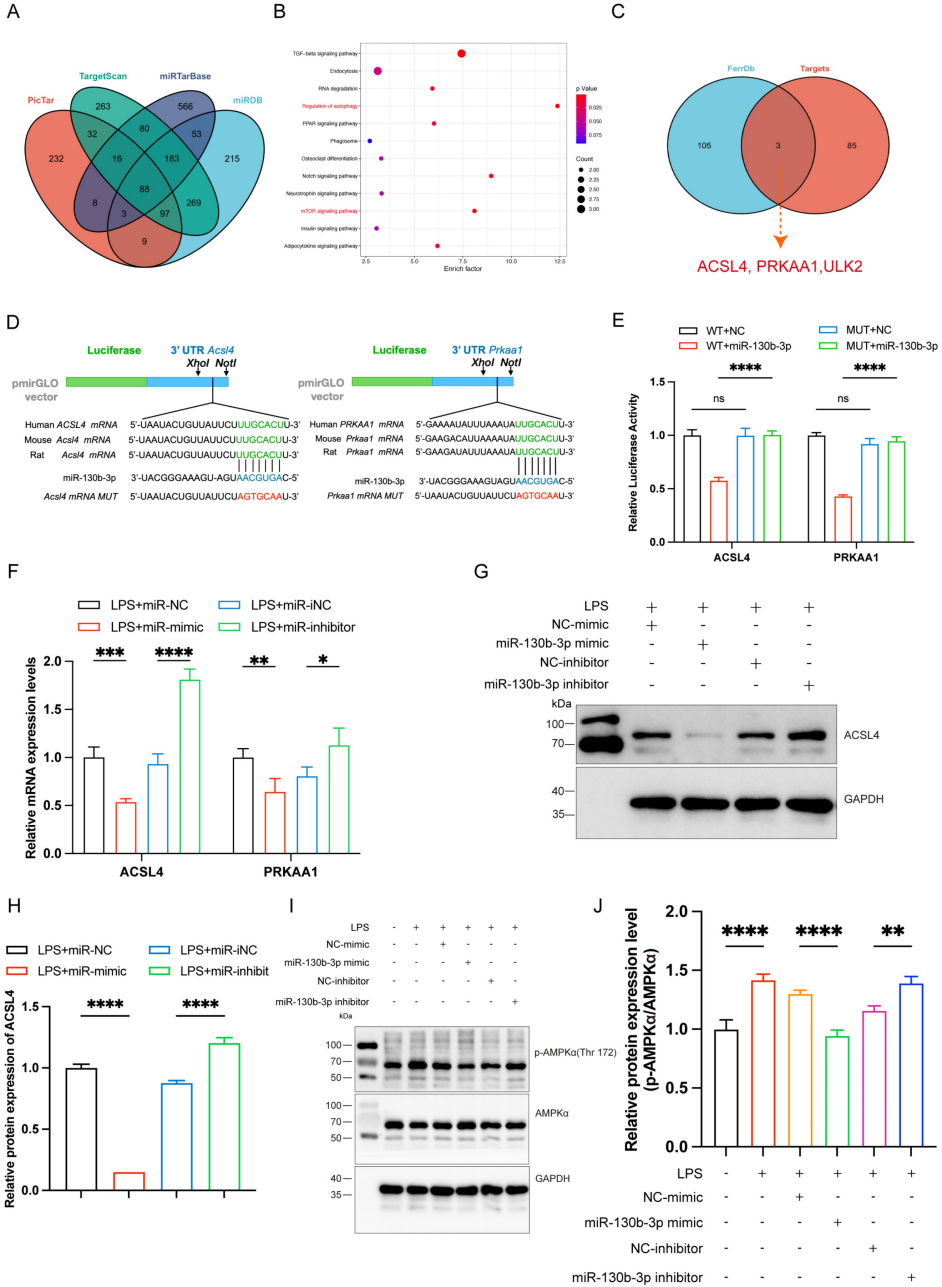
**| ACSL4 and PRKAA1 are the direct miR-130b-3p targets and may mediate its effects on ferroptosis following LPS-induced cardiomyocytes. (A)** The Venn diagram represents the number of transcripts with conserved miR-130b-3p binding sites predicted by PicTar, TargetScan miRTarBase, and miRDB. **(B)** KEGG analysis of the 88 target genes of miR-130b-3p and the Top 12 significant enrichment signaling pathways are shown. **(C)** The Venn diagram represents the intersection of the 88 target genes and the FerrDb database. **(D)** Schematic representation of the predicted binding sites for miR-130b-3p and the mutated binding sites in the 3' UTR of *Acsl4* and *Prkaa1*. **(E)** The H9c2 cells were transfected with dual-luciferase reporter vectors containing the wild-type (WT) or mutant (MUT) *Acsl4* and *Prkaa1* 3' UTR. Then, the H9c2 cells were further transfected with a miR-130b-3p mimic or NC mimic, and luciferase activity was measured. **(F)** The H9c2 cells were transfected with a miR-130b-3p mimic, NC mimic, or miR-130b-3p inhibitor, NC inhibitor for 24 h, then treated with LPS for 12 h. The mRNA expression levels of *Acsl4* and *Prkaa1* were determined by RT-qPCR, n=3. **(G)** Western blot analysis and **(H)**densitometric quantification of the protein expression levels of ACSL4, n=3. **(I)**The protein expression levels of p-AMPKα and AMPKα were detected by Western blot, n=3. **(J)** Densitometric quantification of p-AMPKα and AMPKα, n=3. Data are expressed as mean ± SD. *p < 0.05, **p < 0.01, ***p < 0.001, ****p < 0.0001; ns: no significant difference.

To further elucidate the molecular mechanism of miR-130b-3p regulation of septic cardiomyopathy, we used PicTar (https://pictar.mdc-berlin.de/), TargetScan (http://www.targetscan.org/), miRTarBase, and miRDB (http://www.mirdb.org) target prediction sites for bioinformatic analysis of potential targets of miR-130b-3p. The Venn diagram identified 88 transcripts with conserved miR-130b-3p binding sites (**Figure 5A**). KEGG pathway enrichment analysis of these genes showed that the potential target genes were enriched in the regulation of autophagy and mTOR signaling pathways (**Figure 5B**). Among the 88 predicted targets, only three genes were ferroptosis-related genes according to the FerrDb database (http://www.zhounan.org/ferrdb/current/) (**Figure 5C**). Acyl-CoA synthetase long-chain family member 4 (ACSL4) has been identified as not only a key enzyme that regulates lipid composition but also an important contributor to the execution of ferroptosis 26. Meanwhile, the protein kinase AMP-activated catalytic subunit alpha 1(PRKAA1) has been identified as involved in multiple diseases, including leukemia, heart ischemia/ reperfusion injury in Diabetes, wound healing, and osteoarthritis via autophagy 37, 43-45. Gene prediction tools (TargetScan and miRbase: https://www.mirbase.org/) suggested the presence of a binding site for miR-130b-3p in *Acsl4* and *Prkaa1* that is highly conserved in many species, especially in humans, mice, and rat (**Figure 5D**). Dual luciferase reporter assays showed that miR-130b-3p mimic significantly inhibited the luciferase reporter genes activity of *Acsl4* and *Prkaa1* 3'-UTR WT compared with miR-NC, but did not affect the activity of the *Acsl4* and *Prkaa1* 3'-UTR mutant luciferase reporter genes, indicating that *Acsl4* and *Prkaa1* are the targets of miR-130b-3p (**Figure 5E**). Furthermore, RT-qPCR showed that miR-130b-3p mimic significantly inhibited the mRNA of *Acsl4* and *Prkaa1* compared with miR-NC, while miR-130b-3p inhibitor significantly upregulated the mRNA of *Acsl4* and *Prkaa1* compared with miR-iNC (**Figure 5F**). Western blot showed that miR-130b-3p mimic significantly downregulated the protein expression level of ACSL4 and PRKAA1 compared with miR-NC, while miR-130b-3p inhibitor significantly upregulated the protein expression level of ACSL4 and PRKAA1 compared with miR-iNC (**Figure 5G-J**). The above findings suggest that ACSL4 and PRKAA1 are the direct targets of miR-130b-3p.

### MiR-130b-3p inhibited ferroptosis by targeting ACSL4 in LPS-induced H9c2 cells

**FIGURE 6:**
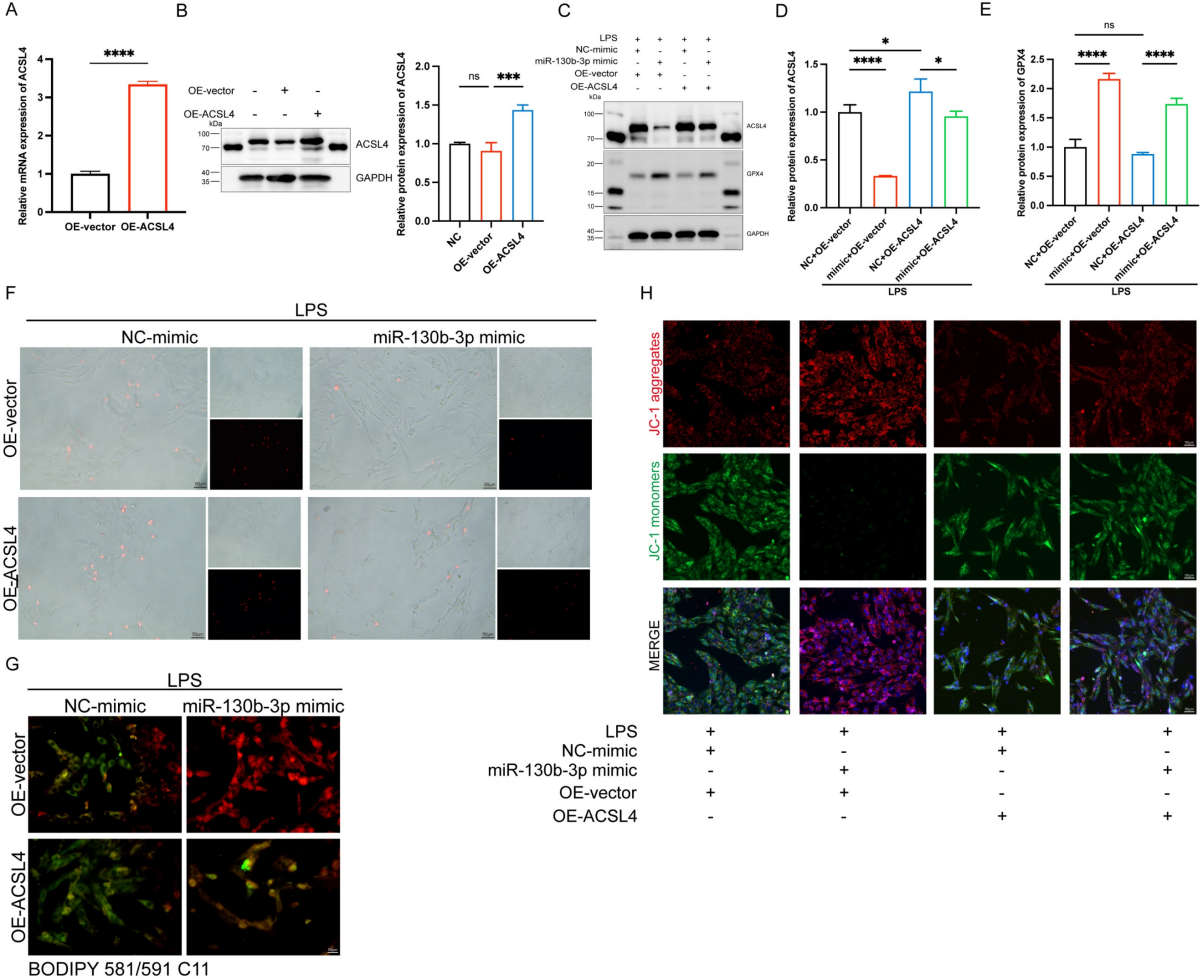
**| miR-130b-3p inhibited ferroptosis by targeting ACSL4 in LPS-induced cardiomyocytes. (A-B)** The H9c2 cells were transfected with OE-vector (overexpressing vector) or OE-ACSL4 (overexpressing *Acsl4* plasmid). **(A)** The mRNA expression levels of *Acsl4* were determined by qRT-PCR, n=3. **(B)** Western blot analysis and densitometric quantification of the protein expression levels of ACSL4, n=3. (C-H) The H9c2 cells were co-transfected with *Acsl4* overexpressing plasmid and miR-130b-3p mimic for 48 h, then treated with LPS for 12h. **(C)** Immunoblots analysis and **(D-E)** densitometric quantification of the protein expression levels of ACSL4 and GPX4 in the indicated groups, n=3. **(F)** Representative images of microscopy (black and white: phase contract; red: PI staining), n=3. Scale bar, 50 μm. **(G)** Representative immunofluorescence staining images of lipid ROS in LPS-induced H9c2 cells via BODIPY 581/591 C11 probe, n=3. Scale bar, 50 μm. **(H)** Representative images of Mitochondrial membrane potential evaluated by JC-1 staining, n=3. Scale bar, 50 μm. Data are expressed as mean ± SD. *p < 0.05, ***p < 0.001, ****p < 0.0001; ns: no significant difference.

To further determine the role of ACSL4 in miR-130b-3p-regulated septic cardiomyopathy, the H9c2 cells were co-transfected with miR-130b-3p mimic and ACSL4 overexpress vector. Our results showed that ACSL4 overexpress vector transfected alone could upregulate the mRNA and protein expression levels of ACSL4 in the H9c2 cells (**Figure 6A, B**). LPS treatment provoked ferroptosis of the H9c2 cells, which were rescued by miR-130b-3p mimic. However, the protective effect of miR-130b-3p was counteracted by the overexpression of ACSL4. As shown in **Figure 6C-E**. LPS treatment significantly increased ACSL4 expression and inhibited GPX4 expression in H9c2 cells, whereas transfected with miR-130b-3p mimics attenuated the LPS-triggered abnormal expression of ferroptosis markers, and the protective effect of miR-130b-3p mimic was reversed by the overexpression of ACSL4. At the same time, we found that miR-130b-3p mimic significantly inhibited LPS-induced cell death (**Figure 6F**), lipid oxidation (**Figure 6G**), and mitochondrial membrane potential abnormal (**Figure 6H**), while the protective effect was reversed by the overexpression of ACSL4. These findings collectively suggest that miR-130b-3p inhibited ferroptosis by targeting ACSL4 in LPS-induced H9c2 cells.

### MiR-130b-3p modulated autophagy by targeting the AMPK/mTOR signaling pathway to alleviate ferroptosis *in vitro* and* in vivo*

**FIGURE 7:**
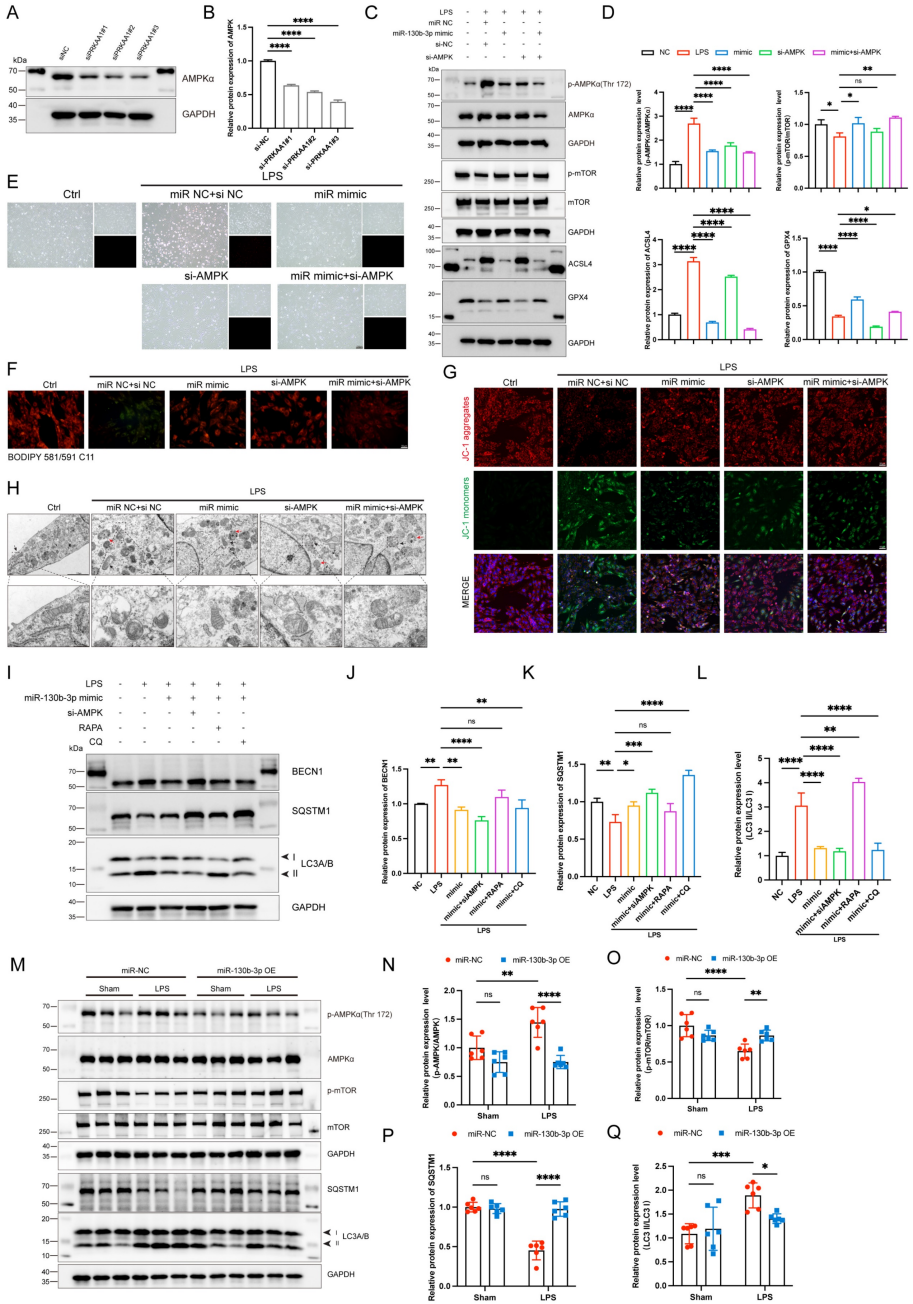
**| miR-130b-3p modulated autophagy by targeting the PRKAA1 to alleviate ferroptosis in LPS-induced cardiomyocytes. (A-B)** The H9c2 cells were transfected with siNC or *Prkaa1* siRNA (siAMPK) for 48 h. **(A)** Western blot analysis and **(B)** densitometric quantification of the protein expression levels of AMPKα, n=3. **(C-D, G-J)** The H9c2 cells were co-transfected with siAMPK and miR-130b-3p mimic for 48 h, then treated with LPS for 12h. **(C)** Immunoblots analysis and **(D)** densitometric quantification of the protein expression levels of p-AMPKα, AMPKα, p-mTOR, mTOR, ACSL4, and GPX4 in the indicated groups, n=3. **(E)** Representative images of microscopy (black and white: phase contract; red: PI staining), n=3. Scale bar, 100 μm. **(F)** Representative immunofluorescence staining images of lipid ROS in LPS-induced H9c2 cells via BODIPY 581/591 C11 probe, n=3. Scale bar, 50 μm. **(G)** Representative images of Mitochondrial membrane potential evaluated by JC-1 staining, n=3. Scale bar, 50 μm. **(H)** Representative TEM images of mitochondria and autophagosome, n=3. Scale bar, 500 nm. Black arrow: mitochondria; Red arrow: autophagosome. **(I-L)** The H9c2 cells were co-transfected with siAMPK and miR-130b-3p mimic for 48 h, then treated with Rapamycin 50 μg/ml for 4 h or Chloroquine 20 μM for 1 h, followed by incubation with LPS for 12 h. **(I)** Immunoblots analysis and** (J-L)** densitometric quantification of the protein expression levels of BECN1, SQSTM1, and LC3 A/B in the indicated groups, n=3. **(M)**The protein expression levels of p-AMPKα, AMPKα, p-mTOR, mTOR, SQSTM1, and LC3 A/B in myocardium from septic mice were detected by Western blot. **(N-Q)** Densitometric quantification of the protein expression levels of p-AMPKα, AMPKα, p-mTOR, mTOR, and SQSTM1 and LC3 A/B in heart tissue of septic mice, n=6. Data are expressed as mean ± SD. *p < 0.05, **p < 0.01, ***p < 0.001, ****p < 0.0001; ns: no significant difference.

To further illustrate the role of PRKAA1 in miR-130b-3p-regulated septic cardiomyopathy, the H9c2 cells were co-transfected with miR-130b-3p mimic and *Prkaa1* siRNA. Western blot showed that *Prkaa1* siRNA transfected alone could downregulate the protein expression levels of PRKAA1 in the H9c2 cells (**Figure 7A, B**). As shown in **Figure 7C, D**, LPS significantly induced the protein expression levels of p-AMPK and ACSL4 and inhibited the expression of p-mTOR and GPX4, while miR-130b-3p reversed the abnormal expression of these proteins. Interestingly, we found that co-transfected with miR-130b-3p mimic and *Prkaa1* siRNA increased the protective effect of miR-130b-3p. Therefore, we hypothesized that miR-130b-3p could regulate the AMPK/mTOR signaling pathway and thereby regulate ferroptosis. Our experimental results showed that LPS treatment induced the H9c2 cell death (**Figure 7E**), lipid peroxidation (**Figure 7F**), and abnormal mitochondrial membrane potential (**Figure 7G**), while miR-130b-3p mimic significantly reversed these adverse damages, and *Prkaa1* siRNA transfection increased the protective effect of miR-130b-3p against LPS-induced ferroptosis *in vitro*. Transmission electron microscopy showed that LPS treatment induced mitochondrial swelling, vacuolar degeneration, mitochondrial cristae rarefication, and increased autophagosome, while miR-130b-3p mimic significantly reversed these unfavorable mitochondrial damages, and *Prkaa1* siRNA increased the protective effect of miR-130b-3p (**Figure 7H**). Western blot showed that LPS treatment significantly upregulated the protein expression levels of autophagy-related markers LC3A/B and BECN1 while downregulated SQSTM1, while miR-130b-3p mimic significantly inhibited the activation of autophagy. Co-transfection with *Prkaa1* siRNA and miR-130b-3p mimic significantly reduced autophagic activity. Meanwhile, our results showed that RAPA administration was able to counteract the inhibitory effect of miR-130b-3p mimic on autophagy, while CQ administration achieved the opposite result *in vitro* (**Figure 7I-L**). As shown in **Figure 7M-Q**, LPS treatment induced the activation of the AMPK/mTOR signaling pathway and upregulated the protein expression level of LC3A/B, and downregulated SQSTM1, while overexpression of miR-130b-3p could significantly reverse this adverse effect *in vivo*. Inversely, knocking down miR-130b-3p activated the AMPK/mTOR signaling pathway and upregulated the protein expression level of LC3A/B, and downregulated SQSTM1 in sepsis-induced cardiomyopathy mice (**Figure S4**). The above results suggest that MiR-130b-3p modulated autophagy by targeting the AMPK/mTOR signaling pathway to alleviate ferroptosis in septic cardiomyopathy.

### The activation of autophagy reversed the protective effect of miR-130b-3p on ferroptosis in LPS-induced septic cardiomyopathy

**FIGURE 8:**
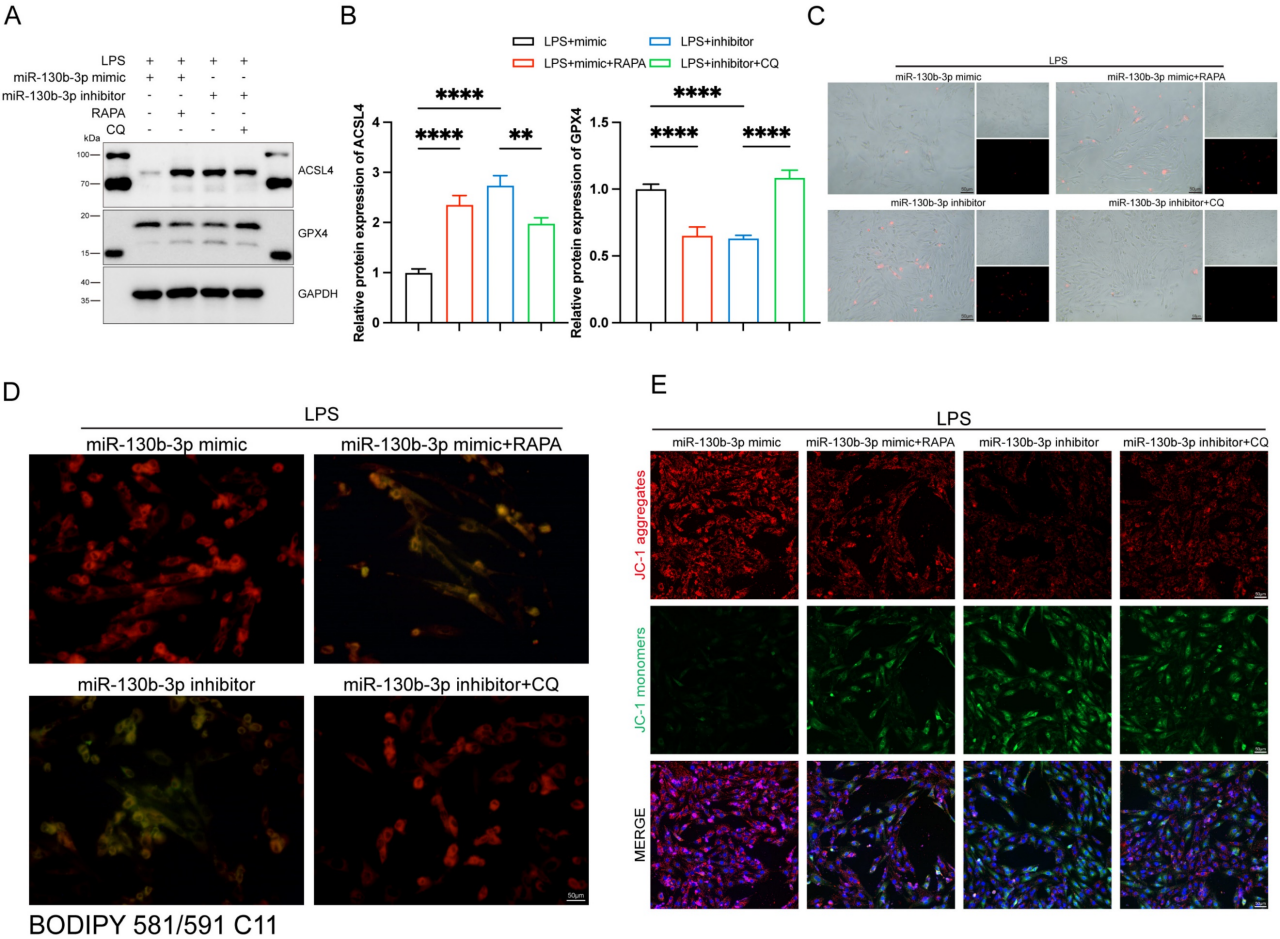
**| Autophagy-modulated ferroptosis in LPS-induced cardiomyocytes. (A-E)** The H9c2 cells were transfected with miR-130b-3p mimic or miR-130b-3p inhibitor for 24 h, then treated with Rapamycin 50 μg/ml for 4 h or Chloroquine 20 μM for 1 h, followed by incubation with LPS for 12 h. **(A)** Western blot was used to analyze the protein expression levels of ACSL4 and GPX4. **(B)** Densitometric quantification of ACSL4 and GPX4, n=3. **(C)** Representative images of microscopy (black and white: phase contract; red: PI staining), n=3. Scale bar, 50 μm. **(D)** Representative immunofluorescence staining images of lipid ROS in H9c2 cells via BODIPY 581/591 C11 probe, n=3. Scale bar, 50 μm. **(E)** Representative images of Mitochondrial membrane potential evaluated by JC-1 staining, n=3. Scale bar, 50 μm. Data are expressed as mean ± SD. **p < 0.01, ****p < 0.0001; ns: no significant difference.

We further investigated the role of autophagy involved in the regulation of ferroptosis in septic cardiomyopathy, the RAPA (an autophagy activator) and CQ (an autophagy inhibitor) were added. The H9c2 cells were transfected with miR-130b-3p mimic and miR-130b-3p inhibitor for 24 h and then treated with Rapamycin 20 μM and Chloroquine 50 μM for 12h in specific groups respectively and then were treated with LPS for 12h. Western blot showed that miR-130b-3p mimic significantly inhibited abnormal ferroptosis-related protein expression induced by LPS, while RAPA administration counteracted the protective effect of miR-130b-3p mimic ferroptosis. On the contrary, the miR-130b-3p inhibitor exacerbated LPS-induced ferroptosis, while CQ administration reversed this adverse effect (**Figure 8A, B**). Meanwhile, we observed by PI staining as well as the white phase that miR-130b-3p mimic attenuated LPS-induced cell death, while RAPA administration counteracted the protective effect. On the contrary, miR-130b-3p inhibitor aggravated LPS-induced H9c2 cell death, while CQ administration attenuated this adverse effect (**Figure 8C**). As shown in (**Figure 8D**), miR-130b-3p mimic attenuated the accumulation of lipid ROS, which was induced by LPS treatment, while RAPA administration exacerbated this detrimental damage. Conversely, miR-130b-3p inhibitor aggravated the accumulation of lipid ROS, while CQ administration alleviated this detrimental damage. JC-1 staining showed that miR-130b-3p mimics significantly improved the decrease in mitochondrial membrane potential levels induced by LPS treatment in H9c2 cells, whereas RAPA treatment reversed the effect of miR-130b-3p mimic. In contrast, miR-130b-3p inhibitor significantly exacerbated the reduction of mitochondrial membrane potential caused by LPS treatment, while administration of CQ alleviated this adverse effect (**Figure 8E**).

## Discussion

Although septic cardiomyopathy is a reversible complication prevalent in patients with sepsis, it has received widespread attention in recent years due to its association with high morbidity and mortality 46. Cardiomyocytes are a class of terminally differentiated cells, and cardiomyocyte death is strongly associated with the prognosis of septic cardiomyopathy, and ferroptosis has been recognized as a protective target against septic cardiomyopathy 19, 30. In the present study, we demonstrated for the first time that miR-130b-3p prevents septic cardiomyopathy by regulating ferroptosis. Overexpression of miR-130b-3p inhibited LPS-induced cardiomyocyte ferroptosis and improved cardiac function in septic cardiomyopathy mice by enhancing mitochondrial function, inhibiting lipid peroxidation, attenuating cardiac pathological injury, and cardiac ultrastructural changes. Mechanistically, miR-130b-3p exerts translational repression by binding to the 3'-UTR region of *Acsl4* and *Prkaa1*. Intriguingly, microRNA-130b-3p exerts a dual function to inhibit ferroptosis in septic cardiomyopathy. miR-130b-3p not only inhibits ferroptosis directly by targeting ACSL4 but also inhibited the AMPK/mTOR signaling pathway to suppress autophagy in cardiomyocytes thereby attenuating ferroptosis, as shown schematically in the **Graphical Abstract**.

Ferroptosis is confirmed as a newly described type of cell death different from other cell death types and is associated with LPS-induced cardiomyopathy 12, 47. Several studies have demonstrated that inhibition of ferroptosis improves cardiomyocyte viability and effectively reduces LPS-induced cardiomyopathy 47-49. Ferroptosis is initiated by severe lipid peroxidation triggered by ROS production and accumulation and iron overload, and reduced expression of antioxidant systems including glutathione GSH and glutathione peroxidase 4 (GPX4) is another crucial trigger 50. However, the literature on the regulatory mechanisms of ferroptosis in sepsis-induced cardiomyopathy is limited and its therapeutic application is still not comprehensively explored. This may be a promising direction to investigate ferroptosis in septic cardiomyopathy.

Recently, non-coding RNAs, especially plasma miRNAs (i.e., miRNA 130b-3p), have gained great recognition as possible functional biomarkers in various clinical situations, including sepsis 41. miRNAs play a vital role as markers of disease progression in multiple physiological and pathological processes 51. To obtain novel biomarkers that serve as prompt indicators of septic cardiomyopathy and screen out the differentially expressed miRNAs (DEMs), we analyzed the GSE174507 of the GEO database. Our data showed that the expression level of miR-130b-3p was significantly downregulated in the Peripheral blood of sepsis patients compared to healthy people. Meanwhile, the expression level of miR-130b-3p was significantly downregulated in the heart tissue of septic cardiomyopathy mice. miR-130b-3p, a member of the miR-130 family, is an evolutionarily conserved small noncoding RNA that has been identified as a key molecule in sepsis that inhibits eCIRP-induced inflammation 41. Therefore, we speculated that miR-130b-3p is involved in septic cardiomyopathy.

In the current study, we found that overexpression of miR-130b-3p improved cardiac functions in septic cardiomyopathy by reducing myocardial histopathological damage, lipid peroxidation, and mitochondrial ultrastructural damage inhibiting ferroptosis* in vivo*. Meanwhile, LPS induced ferroptosis in cardiomyocytes *in vitro*, whereas miR-130b-3p mimic increased cardiomyocyte viability, inhibited lipid peroxidation, improved mitochondrial function, and suppressed ferroptosis. In contrast, treatment with the miR-130b-3p inhibitor significantly aggravated the adverse injury stimulated by LPS in cultured H9c2 cells. These findings are consistent with the previous observation that ferritinophagy-mediated ferroptosis is involved in sepsis-induced cardiac injury 30.

How does miR-130b-3p improve LPS-induced cardiomyopathy? Based on the target genes prediction databases (PicTar, TargetScan, miRTarBase, and miRDB) and FerrDb database, we found that miR-130b-3p could directly target *Acsl4* and *Prkaa1*. Meanwhile, they were confirmed as its target gene by dual luciferase reporter assay. Thus, miR-130b-3p may ameliorate LPS-induced cardiomyopathy by regulating the ACSL4 and AMPK signaling pathways. Acyl-CoA synthetase long-chain family member 4 (ACSL4), which is a key enzyme that regulates lipid composition, has been studied as a crucial factor in metabolism-associated diseases 52. Recently, ACSL4 was found to promote the esterification of arachidonic acid (AA) into phosphatidylethanolamine (PE), a process tightly associated with ferroptosis. Inhibition of ACSL4 through genetics and pharmacology is enabled to suppress this process serving as a rescue pathway against ferroptosis 25. Recent studies have shown that Melatonin downregulated ACSL4 expression to alleviate cardiomyocyte death in doxorubicin-induced ferroptosis 53. Pei et al. demonstrated that FUNDC1 insufficiency induced cardiac remodeling and cardiac contractile dysfunction with short-term high-fat diet (HFD) exposure, likely through ACSL4-mediated regulation of ferroptosis 54. However, the mechanism related to ferroptosis is still uncertain in septic cardiomyopathy. Our study found that miR-130b-3p could directly bind to the 3'UTR region of *Acsl4* to exert translational repression. ACSL4 inhibition increased cell viability, reduced lipid ROS levels, and increased mitochondrial membrane potential in LPS-induced H9c2 cells. Overexpression of ACSL4 by plasmid vector reversed the protective effect of miR-130b-3p on LPS-induced ferroptosis. The PRKAA1 (protein kinase AMP-activated catalytic subunit alpha 1), which belongs to the Ser/Thr protein kinase family, was reported to mediate autophagy in multiple diseases, including diabetes, breast cancer, and cardiac hypertrophy 55-57. However, the previous study shows that there are inconsistent and contradictory results between AMPK expression and ferroptosis 58. Several studies have shown that AMPK activation can improve ferroptosis by inhibiting the function of ACC and decreasing PUFA levels 59-61. In contrast, other studies have shown that AMPK promotes ferroptosis by increasing PUFA levels through the activation of ACSL4 62, 63. This is consistent with our findings that miR-130b-3p can improve ferroptosis by inhibiting the expression levels of AMPK and ACSL4.

Autophagy ensures the normal turnover of organelles and proteins and rapidly removes dysfunctional organelles, dysfunctional mitochondria, and cytotoxic proteins from cells 64. Autophagy can improve myocardial injury in the early stages of septic cardiomyopathy, but continued autophagy can worsen the myocardial injury, so more research is needed to investigate the mechanisms of autophagy in regulating the different stages of myocardial injury 65. However, it was unclear whether the miR-130b-3p could regulate autophagy in septic cardiomyopathy via mediating its target genes. To clarify the specific mechanism, we first examined the expression of related kinases acting upstream of autophagy. AMPK/mTOR is currently recognized as an important molecule in the upstream inhibition of the autophagy formation 66. Our study found that miR-130b-3p reversed the LPS-induced increase in p-AMPKα, and decreased p-mTOR levels. In addition, miR-130b-3p reduced the expression of autophagy-related proteins LC3II and BECN1, indicating that miR-130b-3p inactivated the AMPK/mTOR signaling pathway to inhibit autophagy *in vivo* and *in vitro*. These findings are consistent with the previous reports that ALDH2 protects against LPS-induced cardiomyopathy through a CaMKKβ-AMPK-mTOR mediated regulation of the autophagy 65. Studies have shown that autophagy is activated, leading to lipid peroxidation, mitochondrial injury, and iron accumulation, triggering ferroptosis 12, 67, 68. To confirm that miR-130b-3p regulated LPS-induced ferroptosis by regulating autophagy, we introduced the autophagy activator (RAPA) and autophagy inhibitor (CQ). The levels of ferroptosis-related proteins, lipid peroxidation, and mitochondrial membrane potential were assessed. Our results indicate that RAPA reverses the protective effect of miR-130b-3p mimic on LPS-induced ferroptosis, while CQ ameliorates the promoting effect of the miR-130b-3p inhibitor on LPS-induced ferroptosis, indicating that miR-130b-3p plays an essential role in the development of ferroptosis through the regulation of autophagy *in vitro*.

Despite all these findings, there are still some limitations in our research as follows: 1) It is well known that an individual microRNA may modulate a wide range of transcripts 69. It remains unclear whether miR-130b-3p regulates the expression of detrimental genes having a systemic effect. 2) the mechanism of ACSL4 and PRKAA1 in modulating septic cardiomyopathy is far from elucidated. 3) Using the Recombinant adeno-associated virus gene transfer approach to overexpression of miR-130b-3p suggests that delivery of the therapy may confer protection from septic cardiomyopathy when miR-130b-3p can be directly targeted to the heart; however, a window of at least 21 days is required before increasing miR-130b-3p levels may be effective *in vivo*. Future studies of miR-130b-3p mimics or analogs given at the time of septic cardiomyopathy may elucidate these questions. However, as with all RNA therapies, delivery into cardiac tissue by systemic injection still presents many challenges and requires further study.

## Conclusion

In summary, our study provided novel insight into the pathological mechanisms of sepsis-induced cardiomyopathy, and miR-130b-3p /ACSL4 and miR-130b-3p /AMPK/mTOR signaling may be considered the new targets for the treatment of septic cardiomyopathy. Although our study confirmed miR-130b-3p as an essential factor in attenuating LPS-induced myocardial injury, other target genes of miR-130b-3p or other differentially expressed miRNAs are also necessary to be carefully researched for septic cardiomyopathy. Consequently, miR-130b-3p may be a promising therapeutic strategy or an early biochemical marker for preventing septic cardiomyopathy.

## Supplementary Material

Supplementary figures and tables.
