# Supplementary material for: microRNA-130b-3p Attenuates Septic Cardiomyopathy by Regulating the AMPK/mTOR Signaling Pathways and Directly Targeting ACSL4 against Ferroptosis
Source: Int J Biol Sci. 2023 Aug 15;19(13):4223–41. doi: 10.7150/ijbs.82287 (PMC10496507; doi:10.7150/ijbs.82287)
Supplement: Supplementary file 1 — Supplementary figures and tables. [file ijbsv19p4223s1.pdf]

microRNA-130b-3p Attenuates Septic Cardiomyopathy by Regulating the AMPK/mTOR Signaling Pathways and Directly Targeting ACSL4 against Ferroptosis

Zhen Qi [a, 1], Ruhui Liu [b,f, 1], Haining Ju [c, 1], Mengxi Huang [d], Zhe Li [e], Wei Li [a, **], Yongyi Wang [a, *]

[a] Department of Cardiovascular Surgery, Renji Hospital, School of Medicine, Shanghai Jiao Tong University, Shanghai, China.

[b] Department of Cardiology, West China Second University Hospital, Sichuan University, Chengdu, Sichuan, China.

[c] Department of Cardiology, Seventh People's Hospital of Shanghai University of Traditional Chinese Medicine, Shanghai, China.

[d] Nanjing Drum Tower Hospital Clinical College of Traditional Chinese and Western Medicine, Nanjing University of Chinese Medicine, Nanjing, China.

[e] Department of Critical Care Medicine, Renji Hospital, School of Medicine, Shanghai Jiao Tong University, Shanghai, China.

[f] Cardiology Department of Ministry of Education, Sichuan University, Chengdu, Sichuan, China

* Corresponding author. Department of Cardiovascular Surgery, Renji Hospital, School of Medicine, Shanghai Jiao Tong University, Shanghai, China.

** Corresponding author. Department of Cardiovascular Surgery, Renji Hospital, School of Medicine, Shanghai Jiao Tong University, Shanghai, China.

E-mail address: surlw@126.com (W. Li), wangyongyi@renji.com (Y. Wang).

[1] These authors contributed equally to this work.

Supplementary information

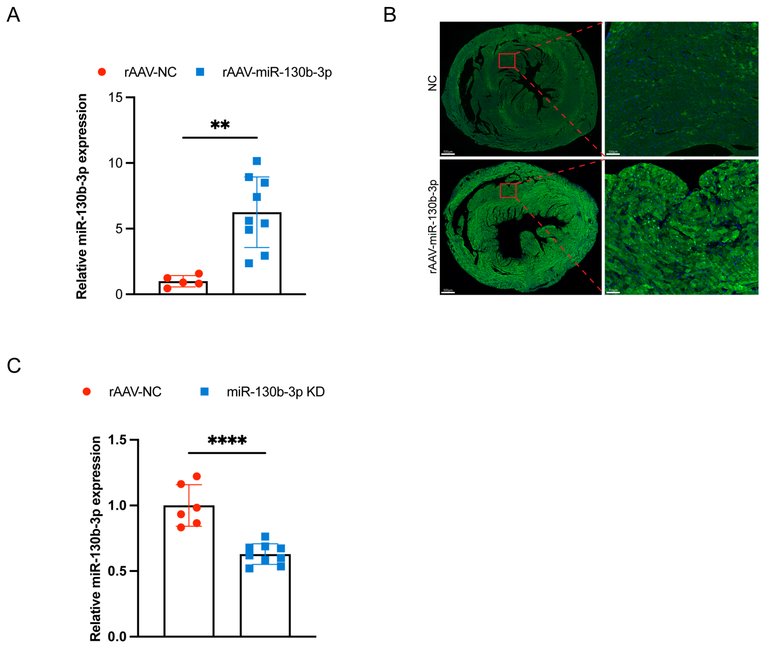

Supplementary Figure S1 (A) qRT-PCR analysis of miR-130b-3p in the myocardium of mice treated with rAAV-miR-NC or rAAV-miR-130b-3p. rAAV-miR-NC group n=5, rAAV-miR-130b-3p group n=9. (B) Representative immunofluorescence staining image of mice hearts treated with rAAV-miR-130b-3p, n=5 per group. (C) qRT-PCR analysis of miR-130b-3p in the myocardium of mice treated with rAAV-miR-NC or rAAV-miR-130b-3p Sponge. rAAV-miR-NC group n=6, rAAV-miR-130b-3p Sponge group n=9. Data are expressed as mean ± SD. **p < 0.01. ****p < 0.0001.

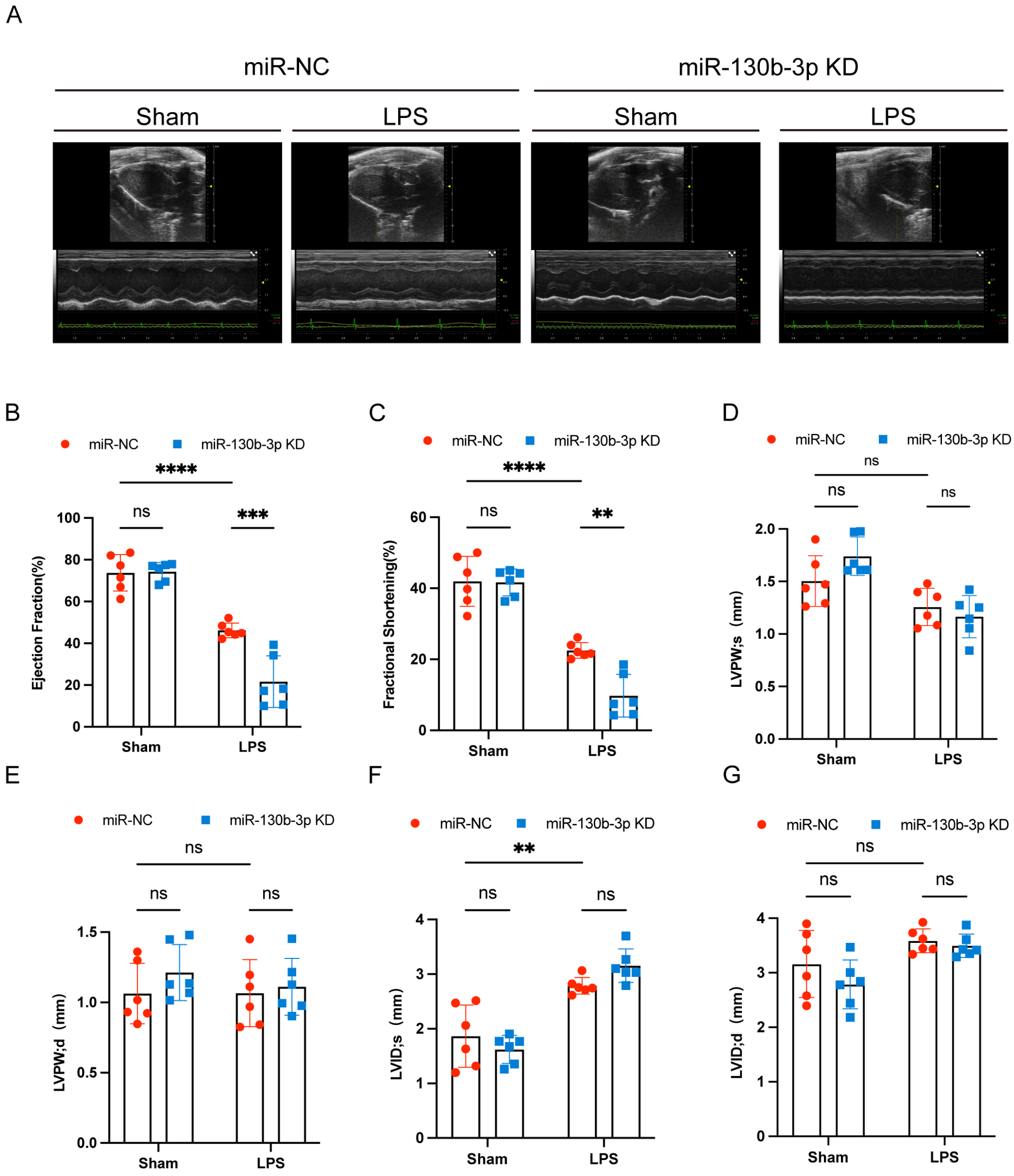

Supplementary Figure S2 | Knocking down miR-130b-3p deteriorated cardiac function in sepsis-induced cardiomyopathy mice. (A) Representative images of echocardiography. (B-G) Echocardiographic analysis of (B) Ejection fraction (EF), (C) Fractional shortening (FS), (D) Left ventricular posterior wall systolic thickness (LVPWs), (E) Left ventricular posterior wall diastolic thickness (LVPWd), (F) Left ventricular internal systolic dimension (LVIDs), and (G) Left ventricular internal diastolic dimension (LVIDd). n=6. Data are expressed as mean ± SD. $^{**}p < 0.01$, $^{***}p < 0.001$, $^{****}p < 0.0001$; ns: no significant difference.

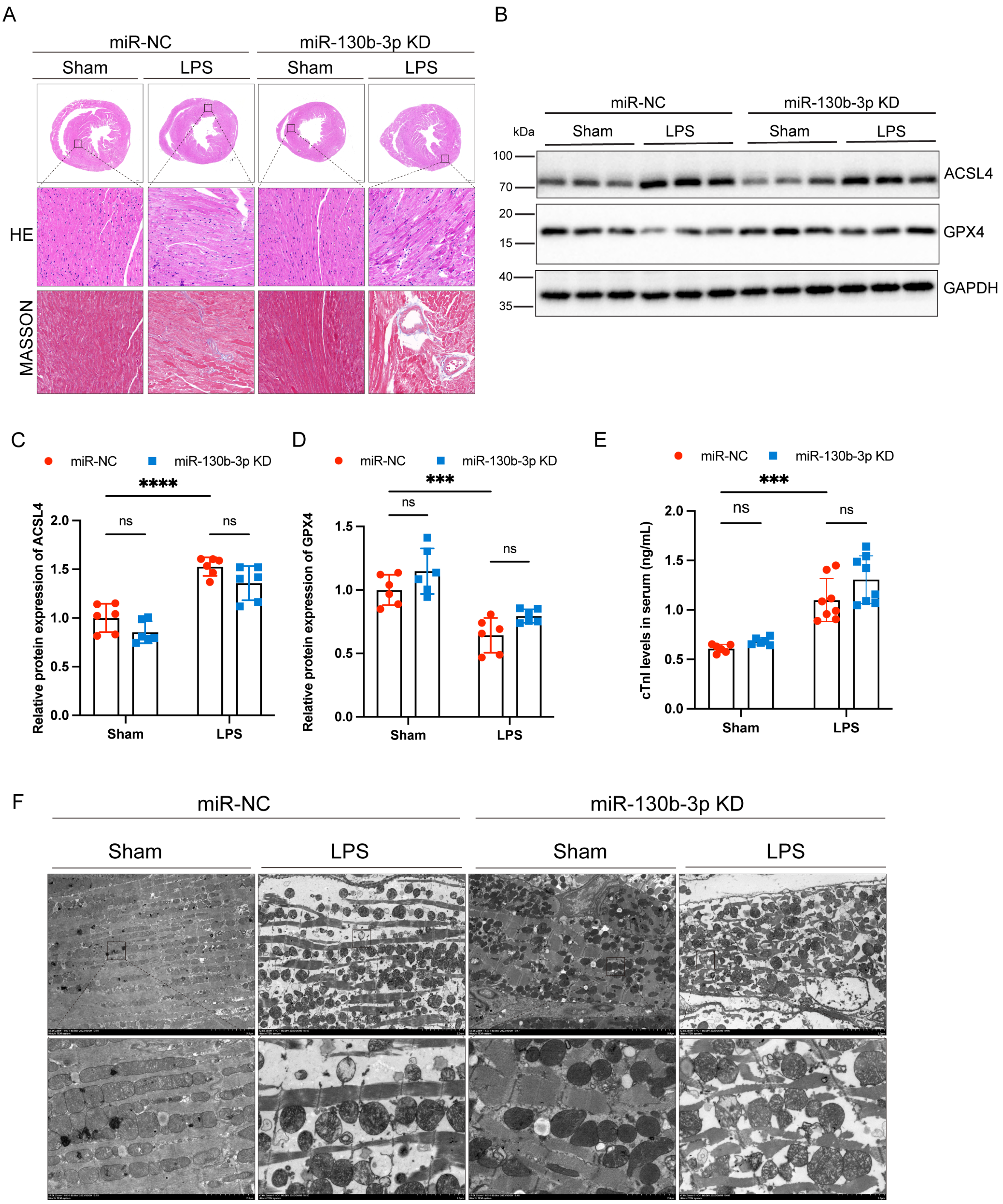

Supplementary Figure S3 | Knocking down miR-130b-3p deteriorated myocardial injury and ferroptosis in sepsis-induced cardiomyopathy mice. (A) Representative H&E staining and Masson trichrome staining images of myocardium from septic mice, n = 6. Scale bar, 20 μm. (B) Representative immunoblots and (C-D) densitometric quantification analysis of the protein expression levels of ACSL4 and GPX4 in cardiac tissue of septic mice, n=6. (E) Serum cardiac troponin (cTnI) levels detected by ELISA, Sham+miR-NC, Sham+miR-130b-3p KD, n = 6; LPS+ miR-NC, LPS+miR-130b-3p

KD, n=8. (F) Representative images of TEM of myocardium from septic mice, n = 6. Scale bar, 2.0 μm. Data are expressed as mean ± SD. **$p < 0.01$, ***$p < 0.001$, ****$p < 0.0001$; ns: no significant difference.

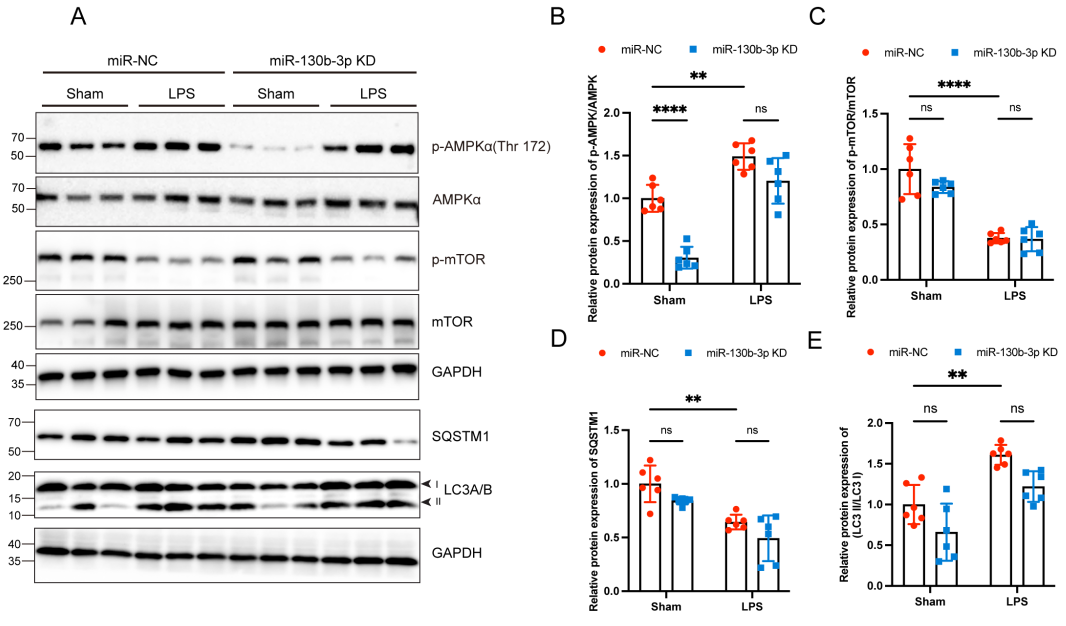

Supplementary Figure S4 | Knocking down miR-130b-3p modulates autophagy by activating the AMPK/mTOR signaling pathway to deteriorate ferroptosis in septic cardiomyopathy. (A) The protein expression levels of p-AMPKα, AMPKα, p-mTOR, mTOR, SQSTM1, and LC3 A/B in myocardium from septic mice were detected by Western blot. (B) Densitometric quantification of the protein expression levels of p-AMPKα, AMPKα, p-mTOR, mTOR, SQSTM1, and LC3 A/B in heart tissue of septic mice, n=6. Data are expressed as mean ± SD. $^{**}p < 0.01$, $^{****}p < 0.0001$; ns: no significant difference.

Supplemental Table S1

The sequences of miRNA mimic, miRNA inhibitor, and small interfering RNA (siRNA)

Gene	Sequences (5′-3′)
rno-miR-130b-3p-mimic	sense: CAGUGCAAUGAUGAAAGGGCAU<br>antisense: AUGCCCUUUCAUCAUUGCACUG
rno-NC-mimic	sense: UUCUCCGAACGUGUCACGU<br>antisense: ACGUGACACGUUCGGAGAA
rno-miR-130b-3p -inhibitor	sense: AUGCCCUUUCAUCAUUGCACUG
rno-NC- inhibitor	antisense: ACGUGACACGUUCGGAGAA
Rat-*Prkaa1*-siRNA#1	sense: GCAGAAGUGUGUAGAGCAATT<br>antisense: UUGCUCUACACACUUCUGCTT
Rat-*Prkaa1*-siRNA#2	sense: CGAGUUGACUGGACAUAAATT<br>antisense: UUUAUGUCCAGUCAACUCGTT
Rat-*Prkaa1*-siRNA#3	sense: GGAUAGUAGGACUUACUUATT<br>antisense: UAAGUAAGUCCUACUAUCCTT
NC-siRNA	sense: UUCUCCGAACGUGUCACGUTT<br>antisense: ACGUGACACGUUCGGAGAATT

Supplemental Table S2 The primer sequences used for quantitative real-time PCR

Gene(miRNA)	Primer (5′-3′)
rno-miR-130b-3p	Forward: GCGCAGTGCAATGATGAAA<br>Reverse: AGTGCAGGGTCCGAGGTATT<br>RT Primer:<br>GTCGTATCCAGTGCAGGGTCCGAGGTATTCGCACTGGA<br>TACGACATGCCC
mmu-miR-130b-3p	Forward: GCGCAGTGCAATGATGAAA<br>Reverse: AGTGCAGGGTCCGAGGTATT<br>RT Primer:<br>GTCGTATCCAGTGCAGGGTCCGAGGTATTCGCACTGGA<br>TACGACATGCCC
U6	Forward: CTCGCTTCGGCAGCACA<br>Reverse: AACGCTTCACGAATTTGCGT<br>RT Primer:<br>GTCGTATCCAGTGCAGGGTCCGAGGTATTCGCACTGGA<br>TACGACAAAATATGGA
Rattus *Acsl4*	Forward: CTCACTGCACTGGGACTGAA<br>Reverse: ATAATGCCGCCTTCAGTTTG
Rattus *Prkaa1*	Forward: TTGCGTGTGCGAAGGAAGAACC<br>Reverse: CTGTGGAGTAGCAGTCCCTGATTTG
Rattus *Gapdh*	Forward: GACATGCCGCCTGGAGAAAC<br>Reverse: AGCCCAGGATGCCCTTTAGT
Mus *Gapdh*	Forward: AAGAGGCTAAGACCGCCTTC<br>Reverse: GCATAAATTCCCACTGCCAC
